# Supplementary material for: Discovery of functional factorless internal ribosome entry site-like structures through virome mining
Source: PLoS Pathog. 2025 Jun 26;21(6):e1013255. doi: 10.1371/journal.ppat.1013255 (PMC12221177; doi:10.1371/journal.ppat.1013255)
Supplement: S6 Data — (PDF) [file ppat.1013255.s006.pdf]

Extended S6 Data

SAXS analysis of non-DV-1 IRES

SAXS is a biophysical method that offers low-resolution structural information about the shape and conformation of RNA in solution [1,2]. The raw data for non-DV-1 RNA (Fig. S5A-S5D) produced Guinier, Kratky, and P(r) plots. The Guinier analysis was applied to evaluate the quality of the collected data and determine the radius of gyration (R_g) (Fig. S5D). The plots were subjected to linear regression fitting, and the observed linearity indicates that the samples are devoid of aggregation and are monodisperse (Fig. S5B), with an R_g of approximately 55.84 Å. We then used a dimensionless Kratky analysis to assess the compactness of each molecule. Upon reaching a near-maxima on the dimensionless Kratky distribution, the data set exhibits a plateau-like distribution (Fig. S5C), signifying an extended conformation for the RNAs. Finally, we constructed a paired electron distribution function to calculate reciprocal-space R_g and their maximum dimensions (D_{max}). The R_g calculated from the P(r) function (56.07 Å) agrees with Guinier R_g (55.84 Å). Also, the shape of the P(r) curves from symmetrical Gaussian distributions supports the Kratky analysis, indicating that an elongated molecule is under examination (Fig. S5D). Furthermore, it is evident from the P(r) that the non-DV-1 RNA has a D_{max} of approximately 175 Å. DAMMIN was employed to perform ab initio modelling and produce low-resolution envelope structures for non-DV-1 RNAs using the P(r) information.

References

1. D'Souza MH, Mrozowich T, Badmalia MD, Geeraert M, Frederickson A, Henrickson A, et al. Biophysical characterisation of human LincRNA-p21 sense and antisense Alu inverted repeats. Nucleic Acids Res. 2022;50: 5881–5898. doi:10.1093/nar/gkac414

2. Chen Y, Chapagain S, Chien J, Pereira HS, Patel TR, Inoue-Nagata AK, et al. Factor-Dependent Internal Ribosome Entry Site and -1 Programmed Frameshifting Signal in the Bemisia-Associated Dicistrovirus 2. Viruses. 20240428th ed. 2024;16. doi:10.3390/v16050695
