# Supplementary figures and images for: Discovery of functional factorless internal ribosome entry site-like structures through virome mining
Source: PLoS Pathog. 2025 Jun 26;21(6):e1013255. doi: 10.1371/journal.ppat.1013255 (PMC12221177; doi:10.1371/journal.ppat.1013255)

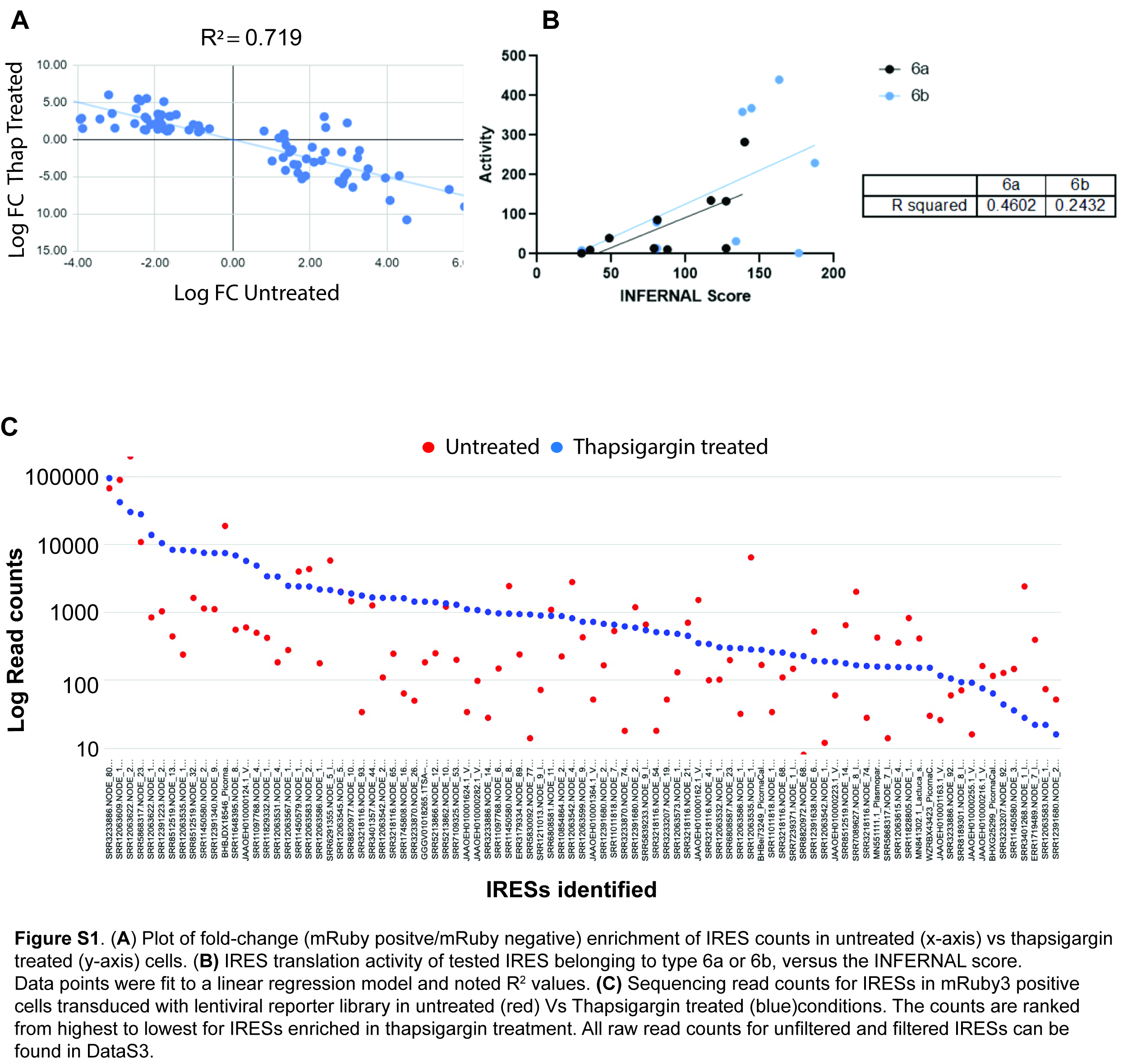

Supplement: S1 Fig — (TIF) [file ppat.1013255.s007.tif]

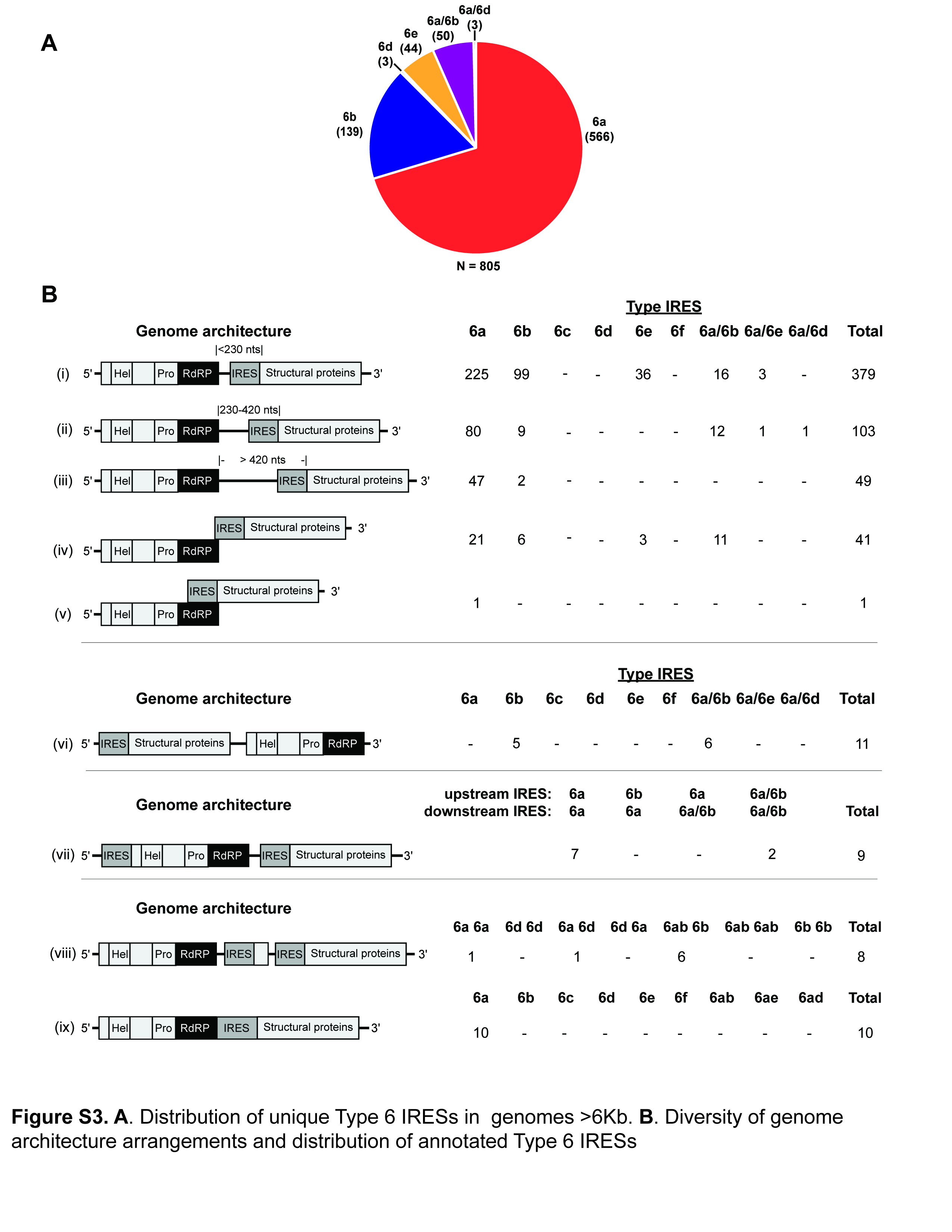

Supplement: S3 Fig — (TIF) [file ppat.1013255.s009.tif]

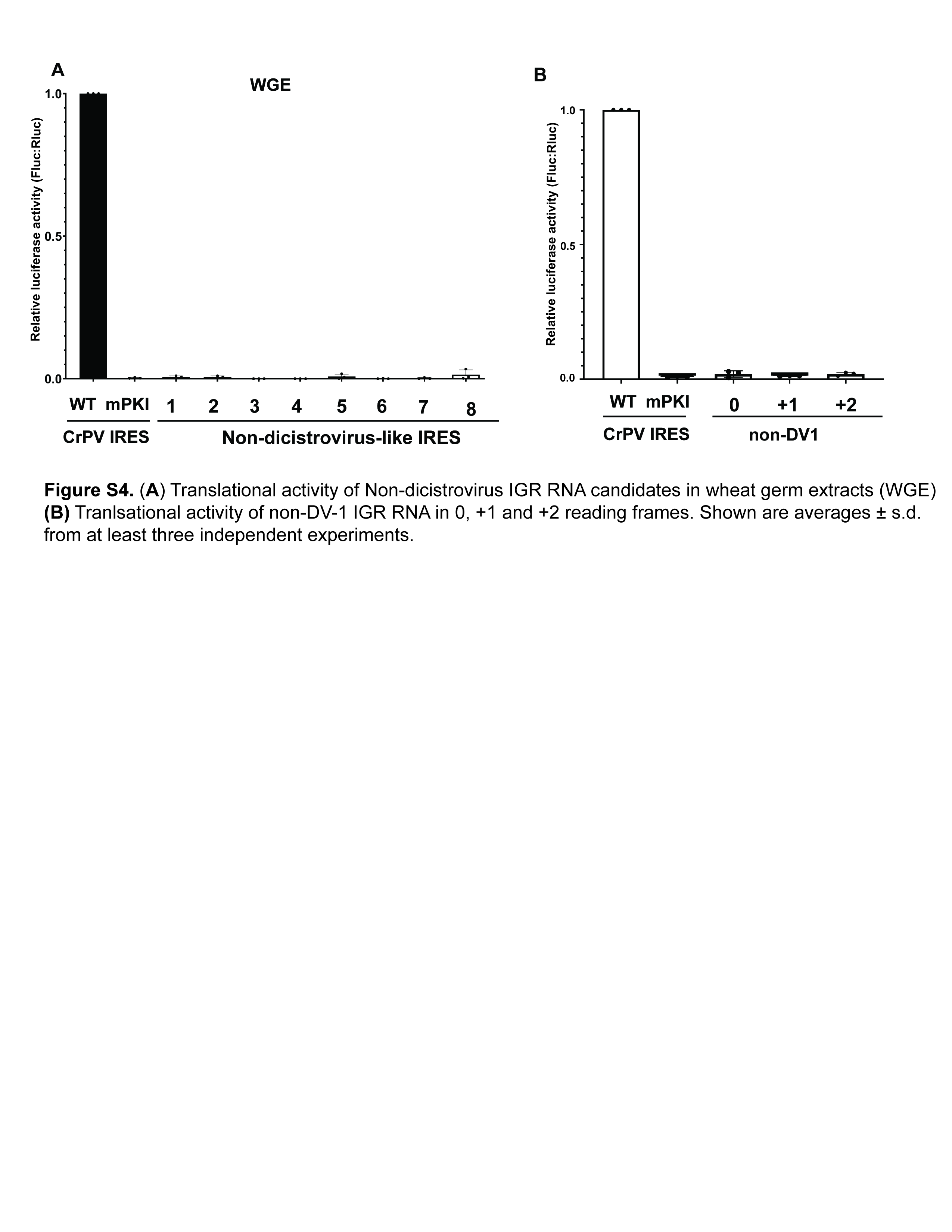

Supplement: S4 Fig — (TIF) [file ppat.1013255.s010.tif]

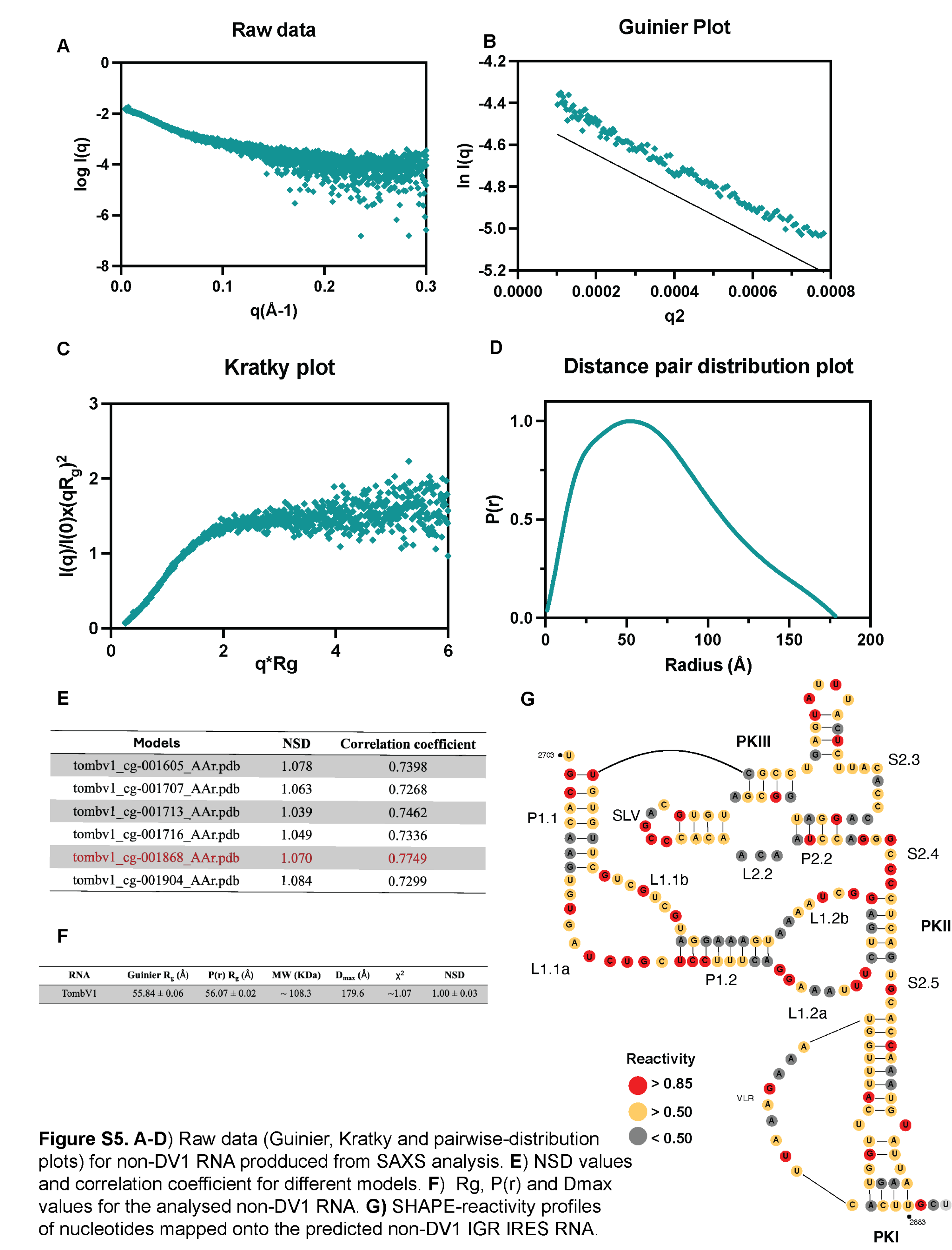

Supplement: S5 Fig — Detailed description of SAXS analysis. (TIF) [file ppat.1013255.s011.tif]
