# Supplementary material for: Discovery of functional factorless internal ribosome entry site-like structures through virome mining
Source: PLoS Pathog. 2025 Jun 26;21(6):e1013255. doi: 10.1371/journal.ppat.1013255 (PMC12221177; doi:10.1371/journal.ppat.1013255)
Supplement: S2 Fig — (PDF) [file ppat.1013255.s008.pdf]

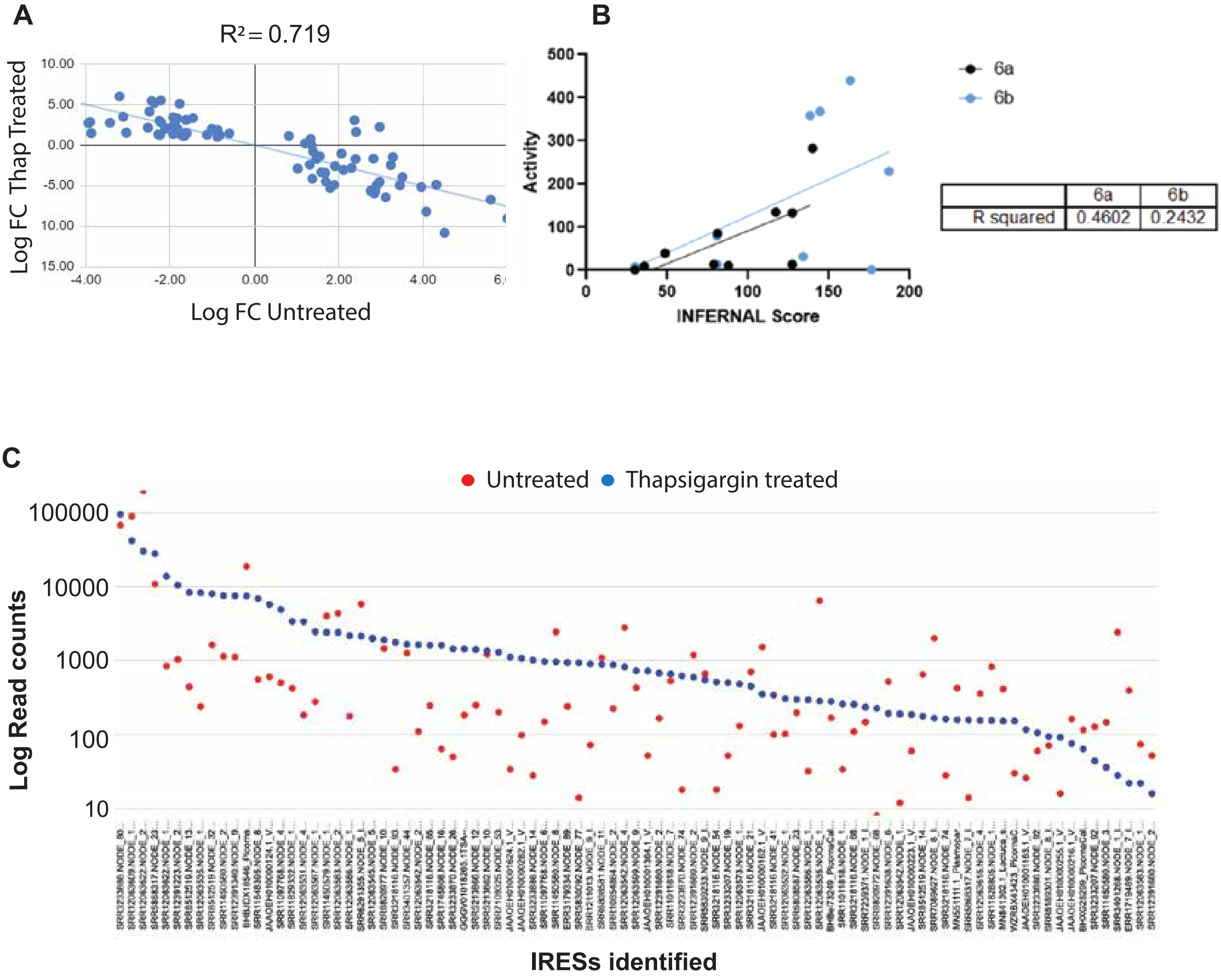

Figure S1. (**A**) Plot of fold-change (mRuby positve/mRuby negative) enrichment of IRES counts in untreated (x-axis) vs thapsigargin treated (y-axis) cells. (**B)** IRES translation activity of tested IRES belonging to type 6a or 6b, versus the INFERNAL score. Data points were fit to a linear regression model and noted R^2 values. **(C)** Sequencing read counts for IRESs in mRuby3 positive cells transduced with lentiviral reporter library in untreated (red) Vs Thapsigargin treated (blue)conditions. The counts are ranked from highest to lowest for IRESs enriched in thapsigargin treatment. All raw read counts for unfiltered and filtered IRESs can be found in DataS3.

Supplemental Figure S2

Type 6a IGR IRESs

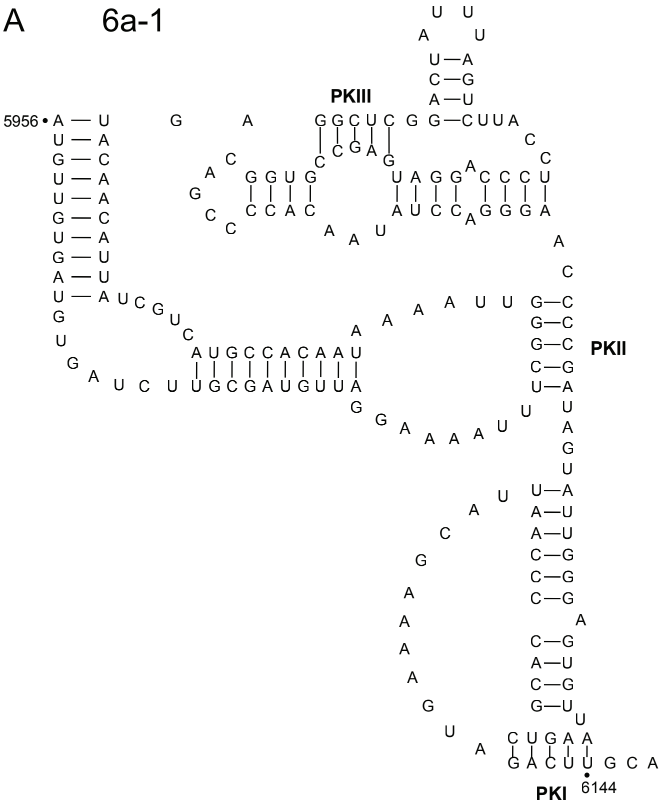

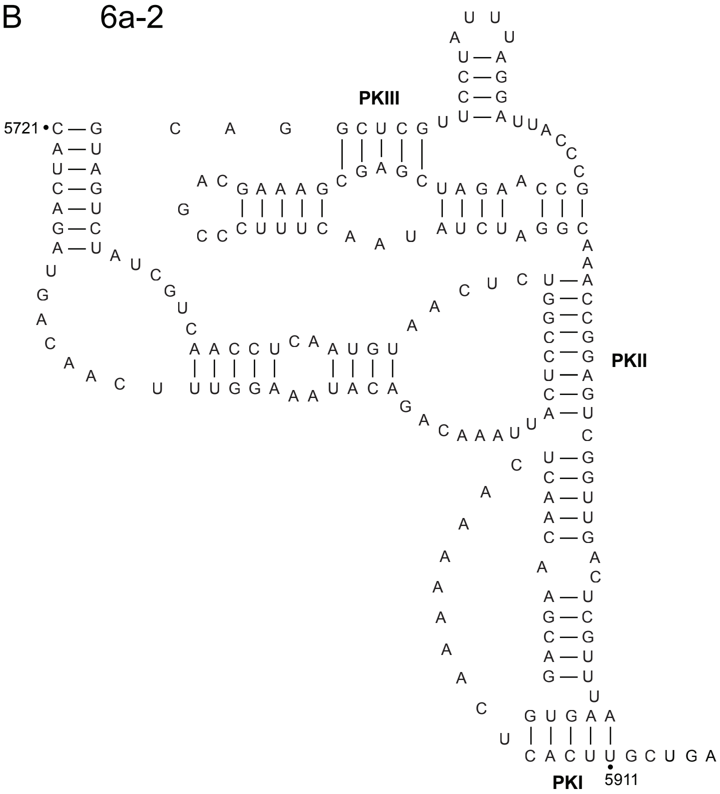

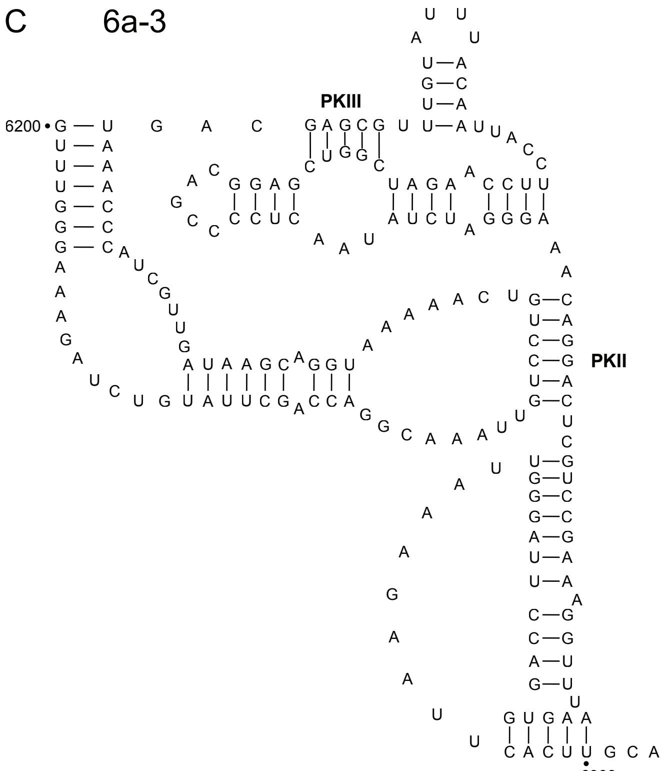

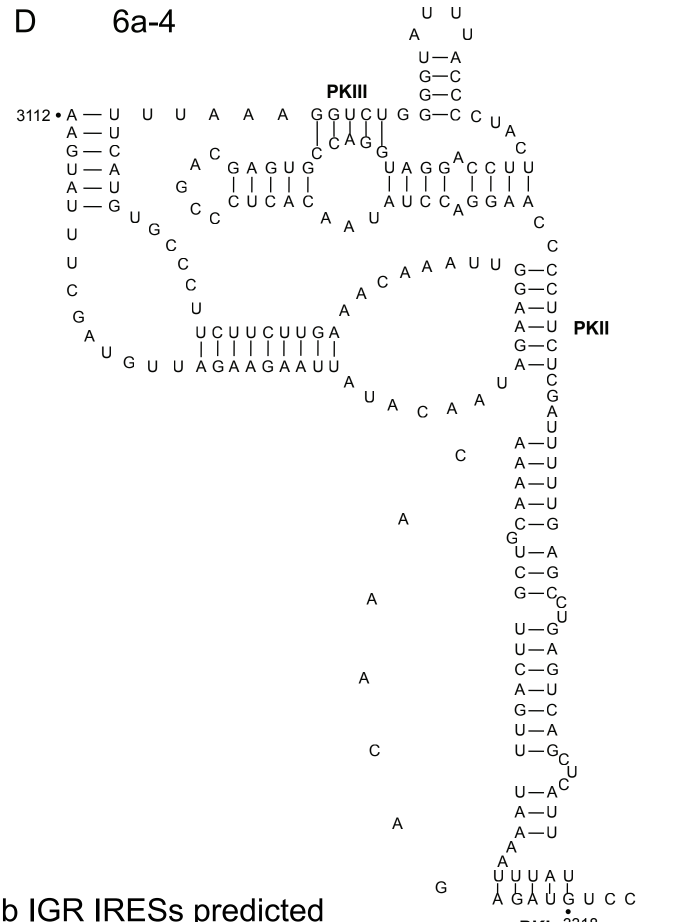

Figure S2. Secondary structure models of Type 6a and 6b IGR IRESs predicted by INFERNAL. INFERNAL scores and sequences are listed in Supplemental Data S4.

E 6a-5

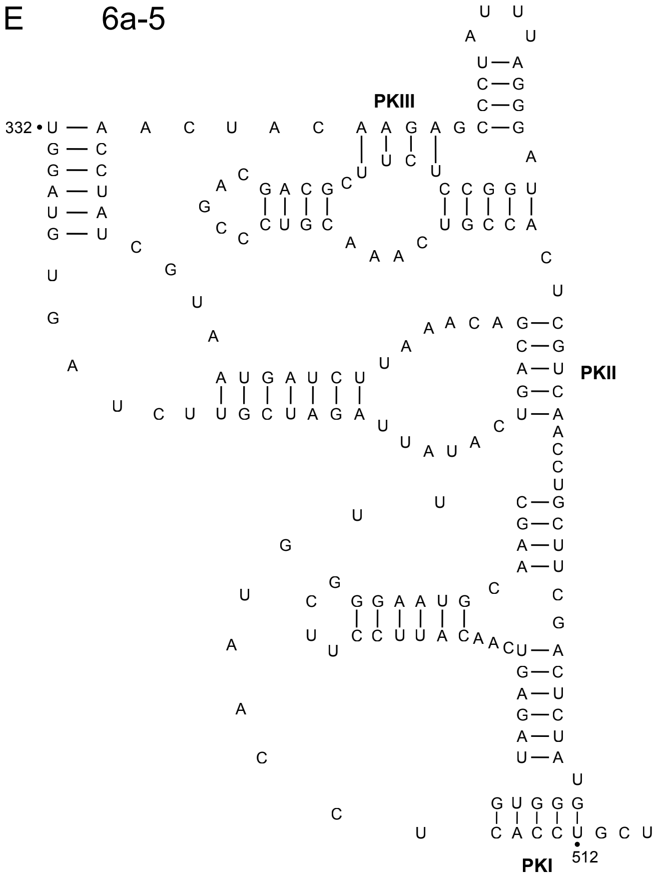

F 6a-6

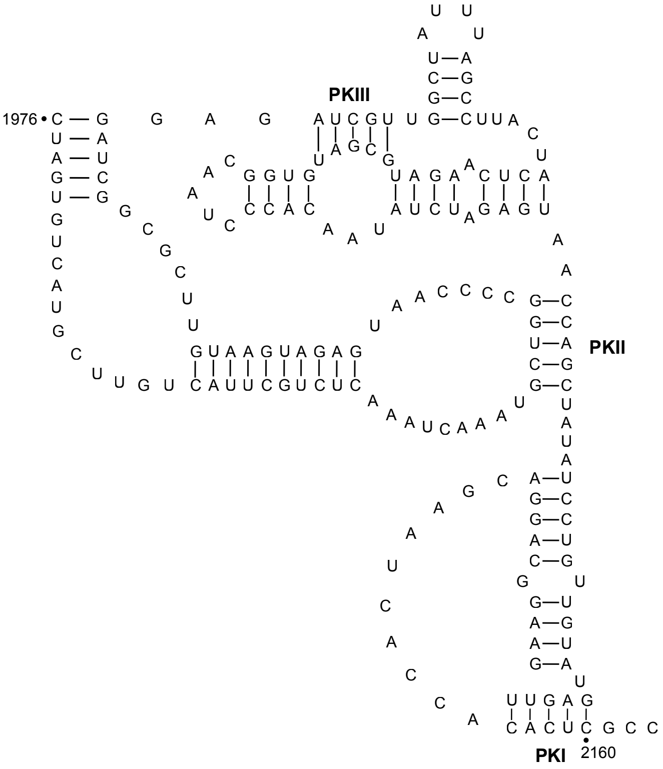

G 6a-7

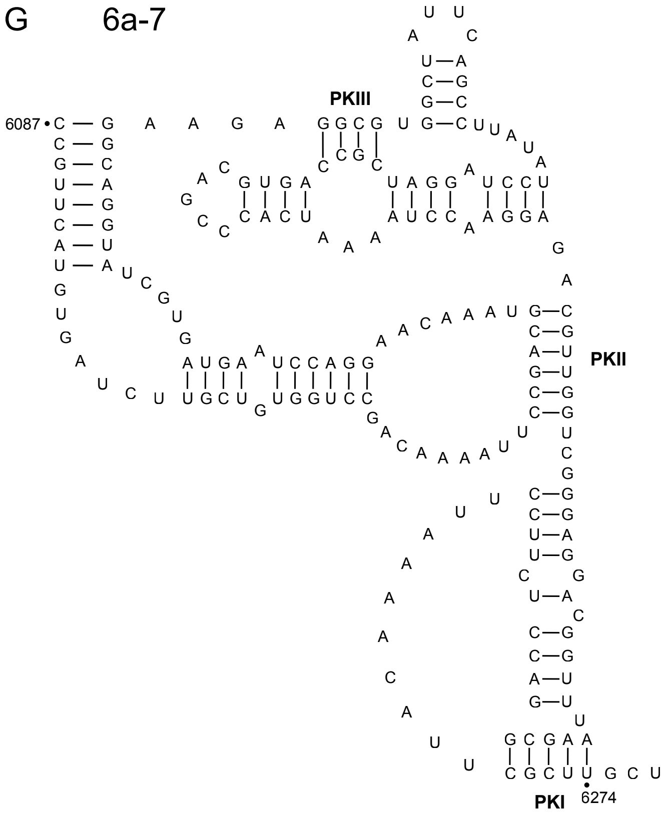

H 6a-8

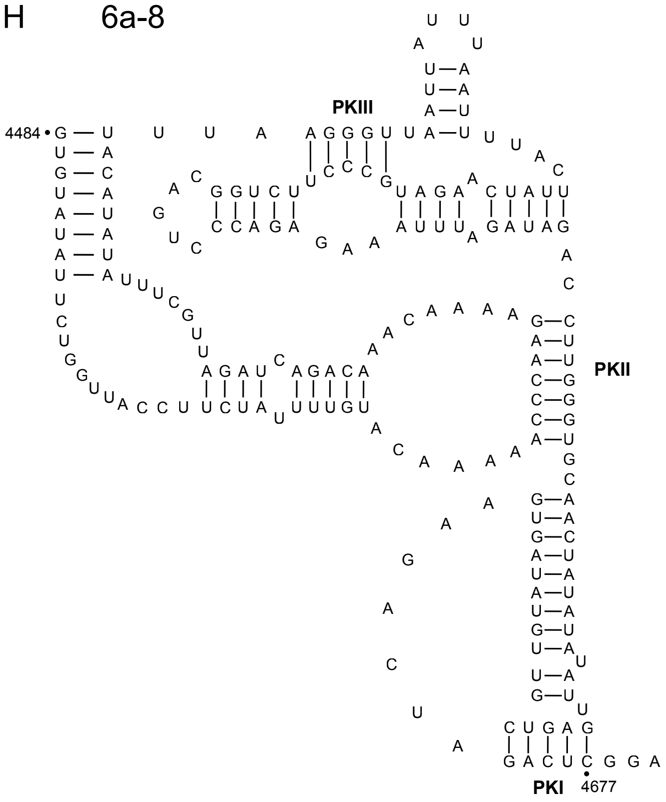

I 6a-9

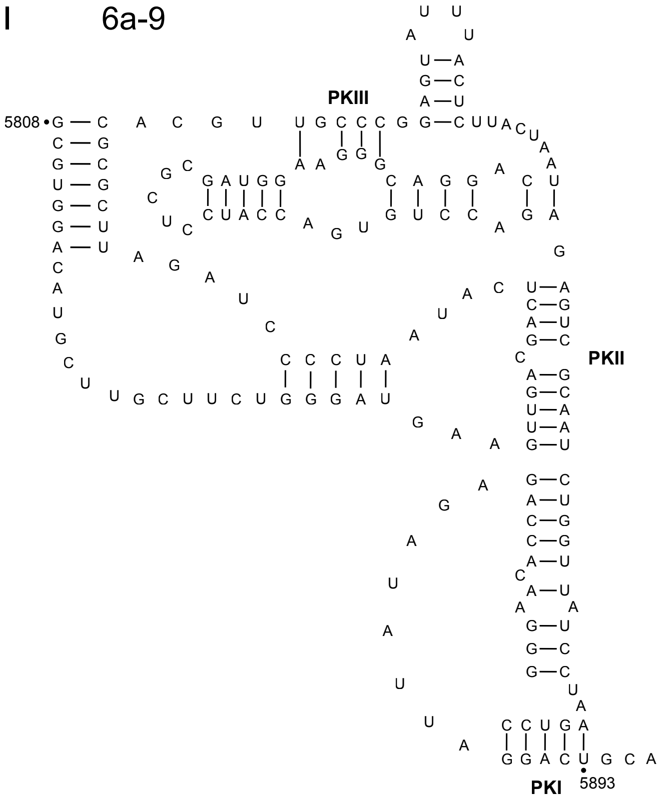

J 6a-10

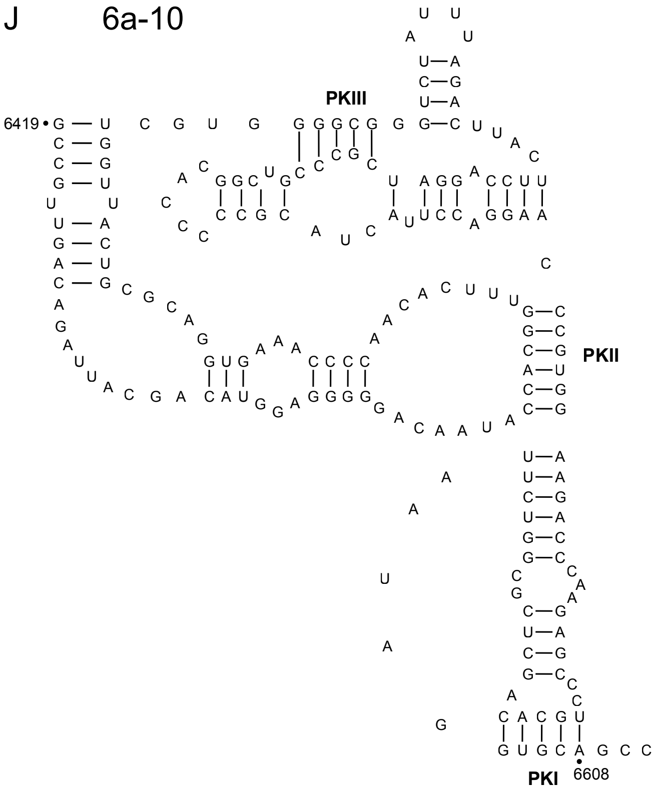

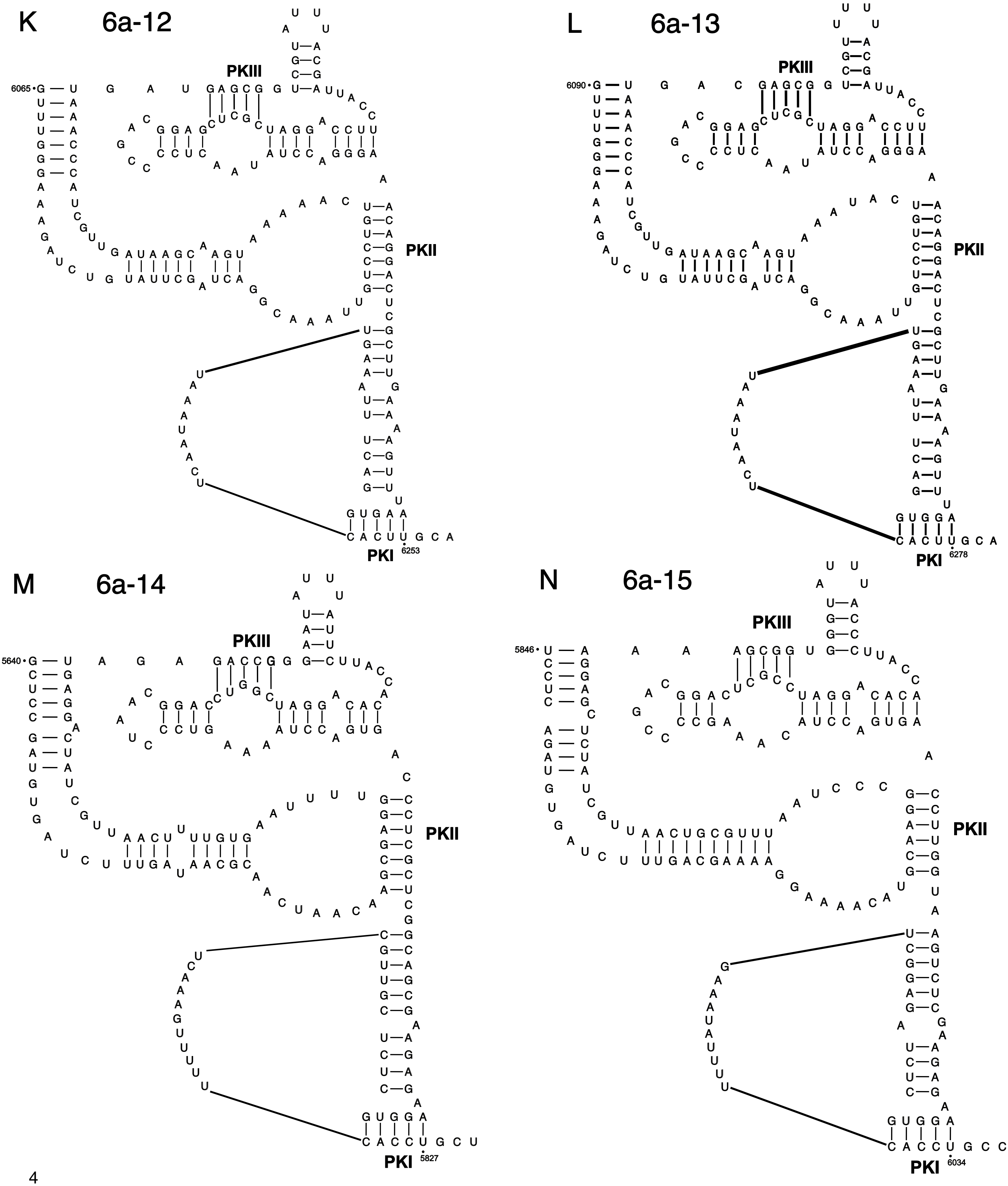
K
6a-12
PKIII
PKII
PKI
6065
6253
L
6a-13
PKIII
PKII
PKI
6090
6278
M
6a-14
PKIII
PKII
PKI
5640
5827
N
6a-15
PKIII
PKII
PKI
5846
6034

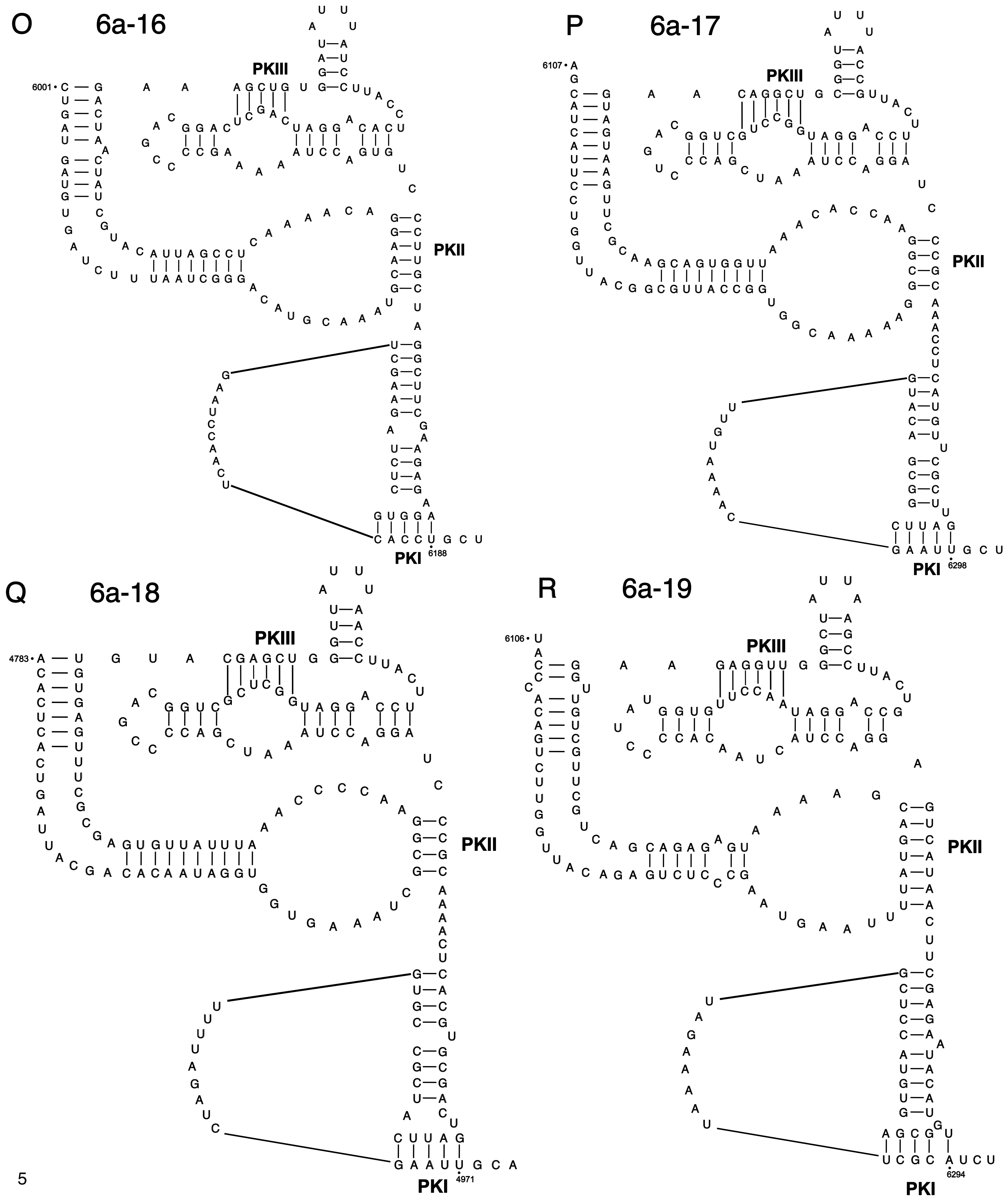

O
6a-16
PKIII
PKII
PKI
6001
6188
P
6a-17
PKIII
PKII
PKI
6107
6298
Q
6a-18
PKIII
PKII
PKI
4783
4971
R
6a-19
PKIII
PKII
PKI
6106
6294

Type 6b IGR IRESs

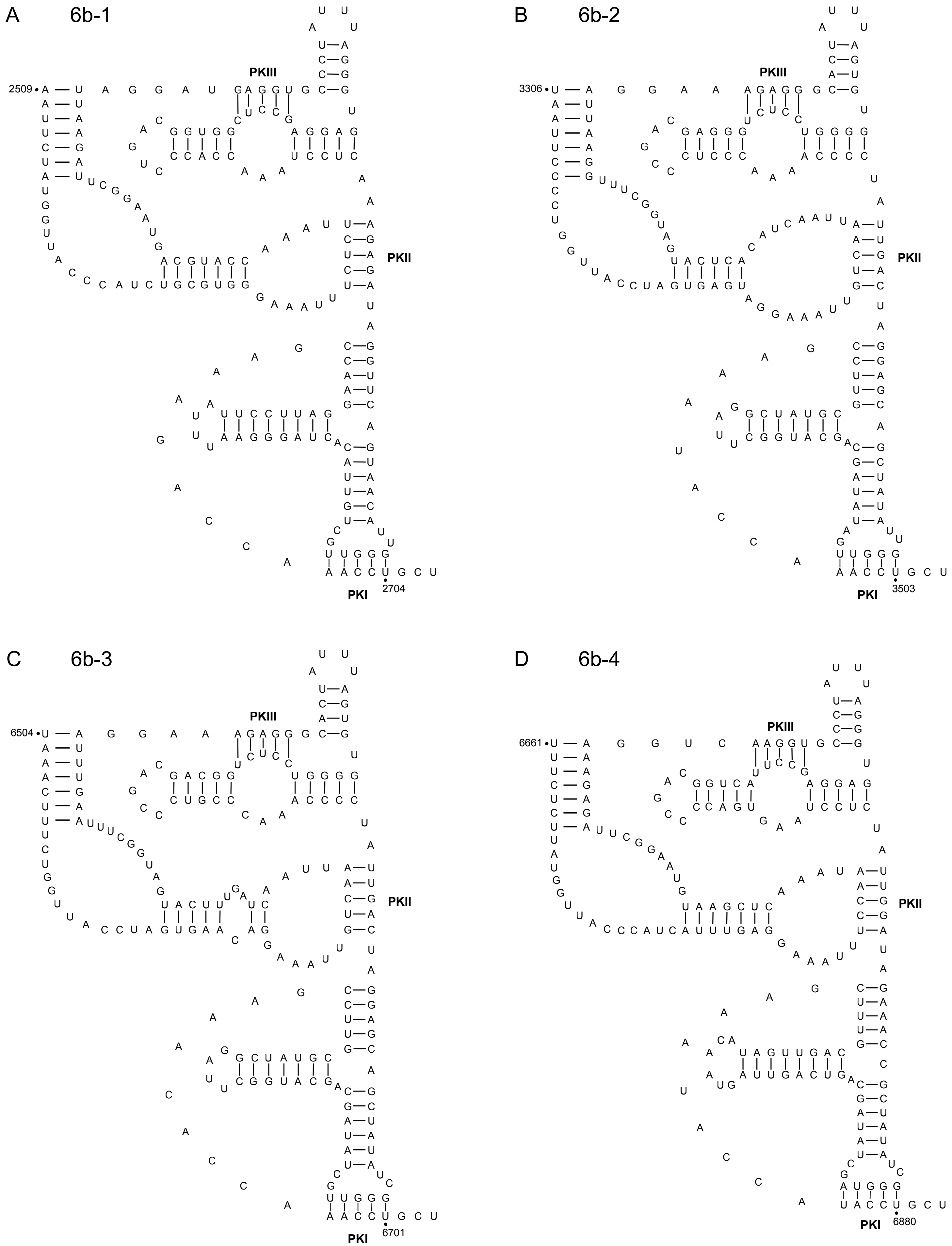

E 6b-5

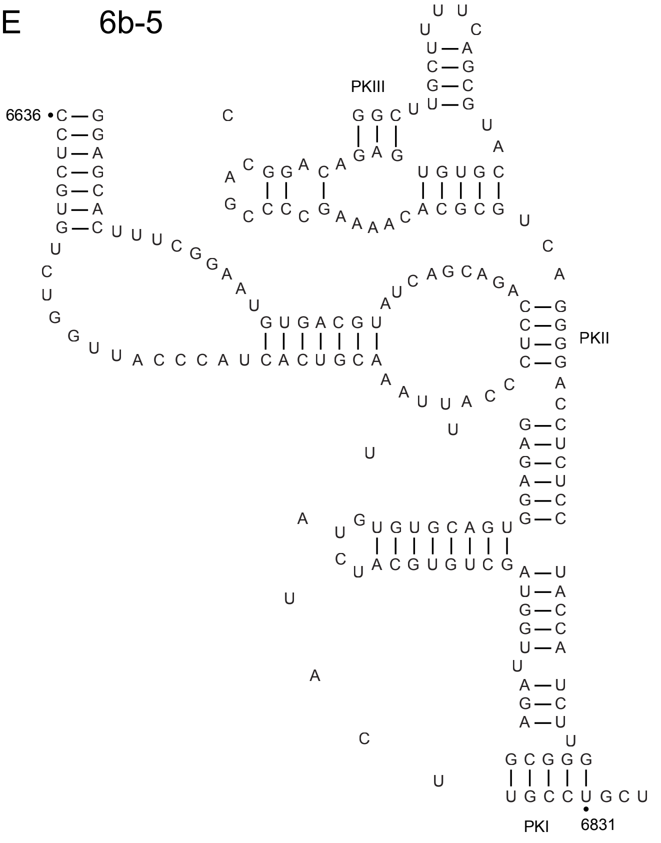

F 6b-6

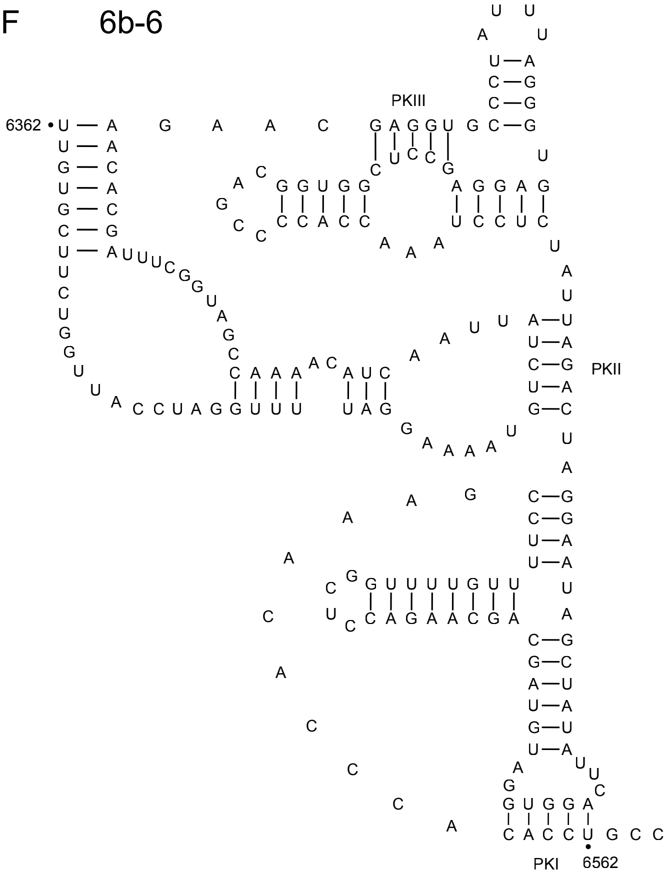

G 6b-7

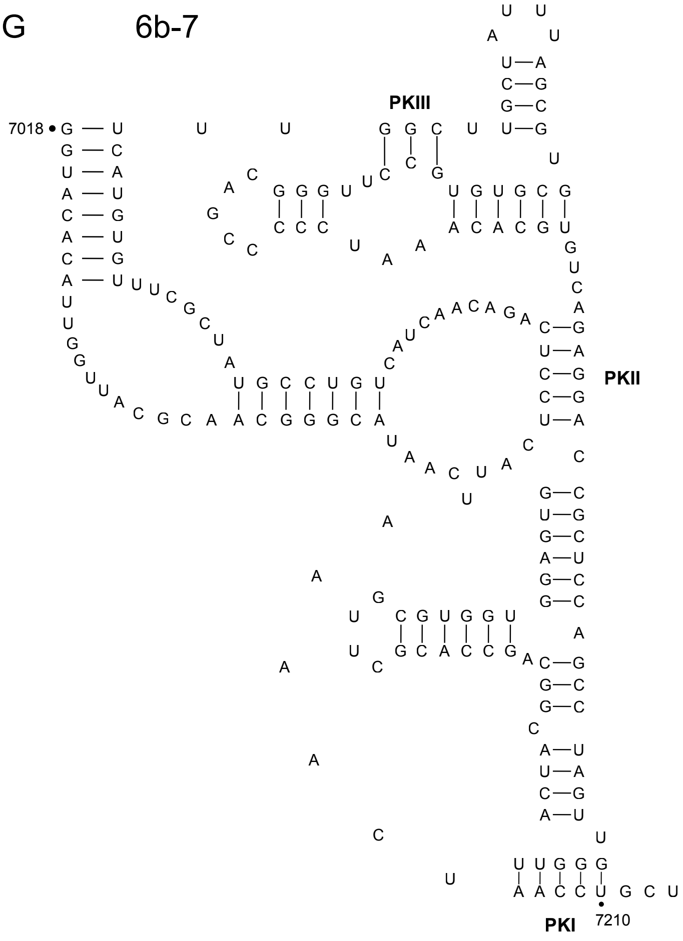

H 6b-8

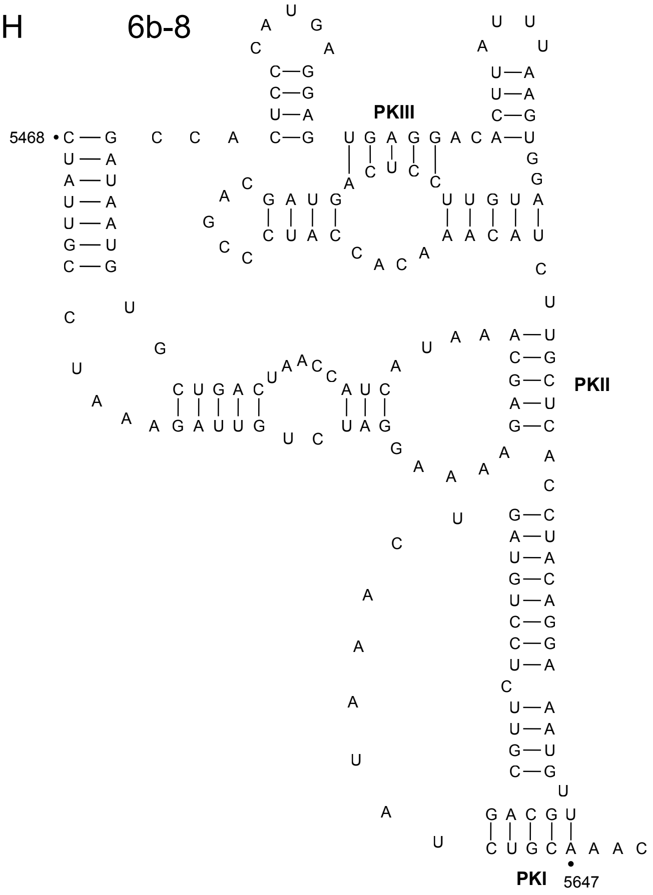

I 6b-9

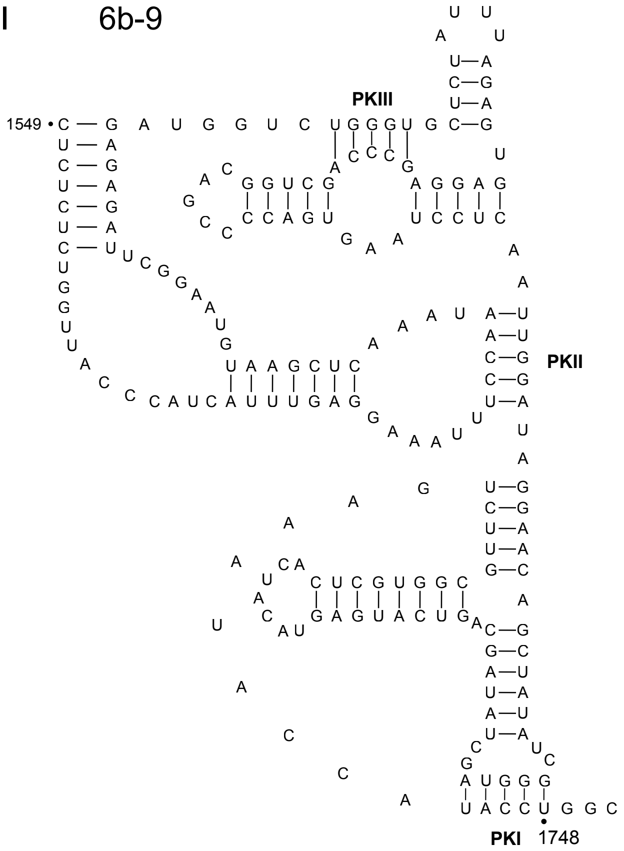

J 6b-10

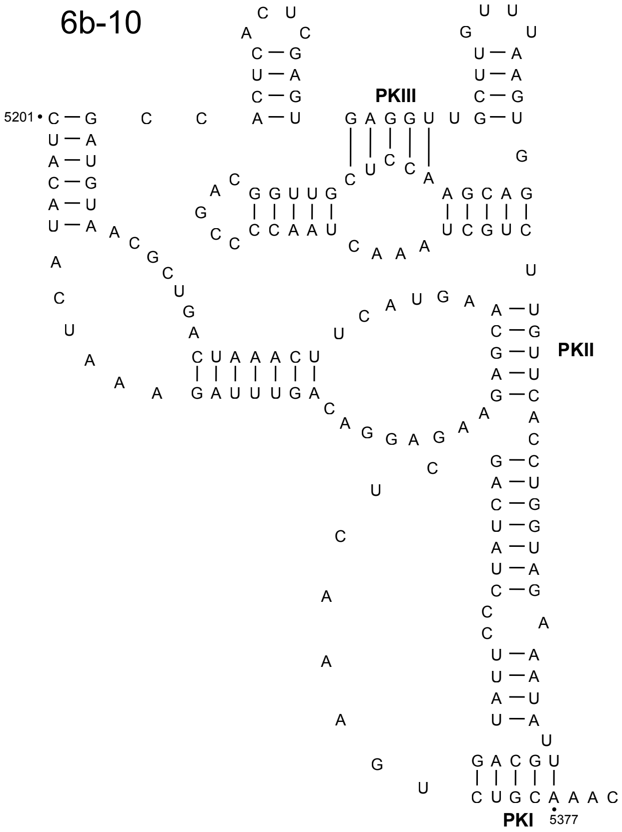

K 6b-11

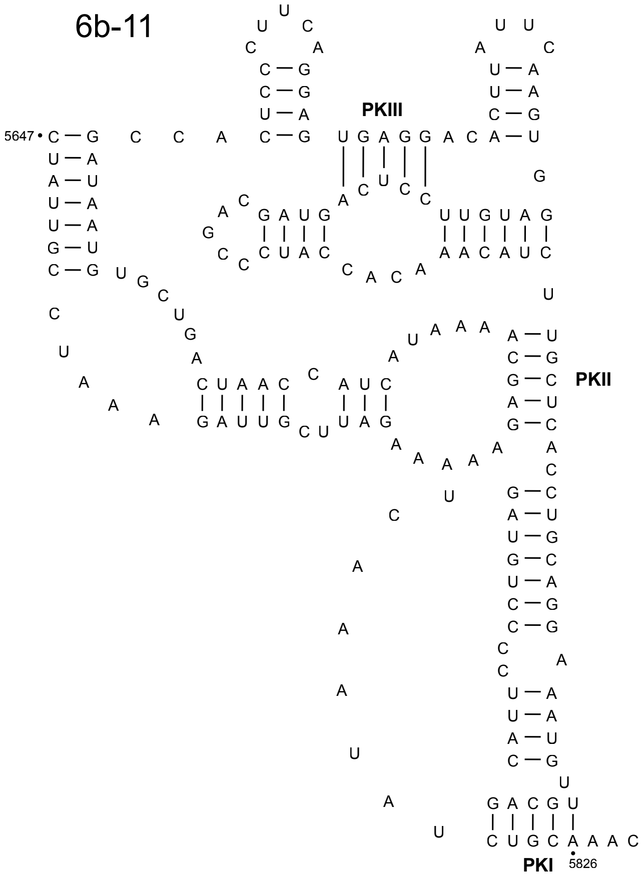

non-Dicistrovirus (n-DV) IGR IRESs

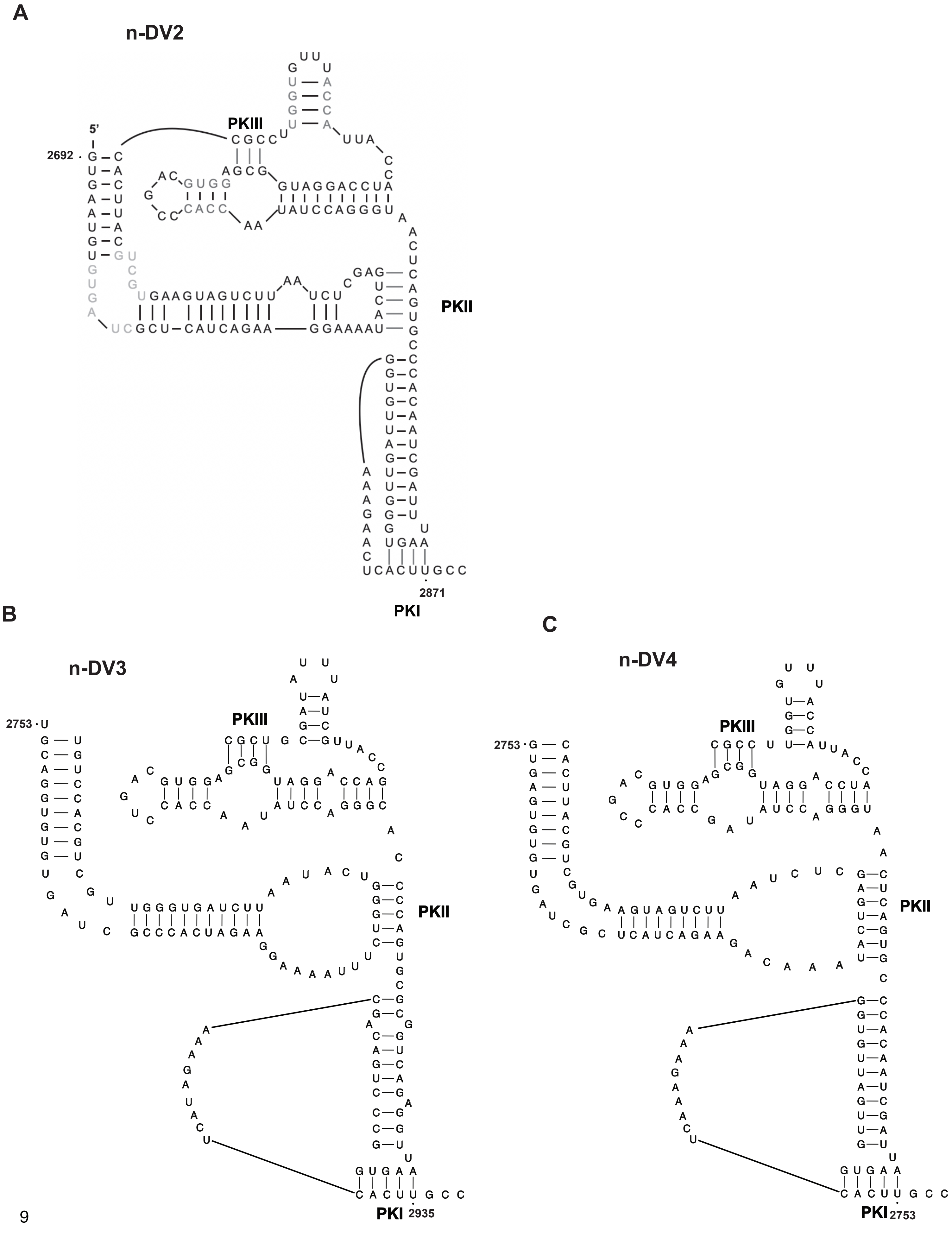

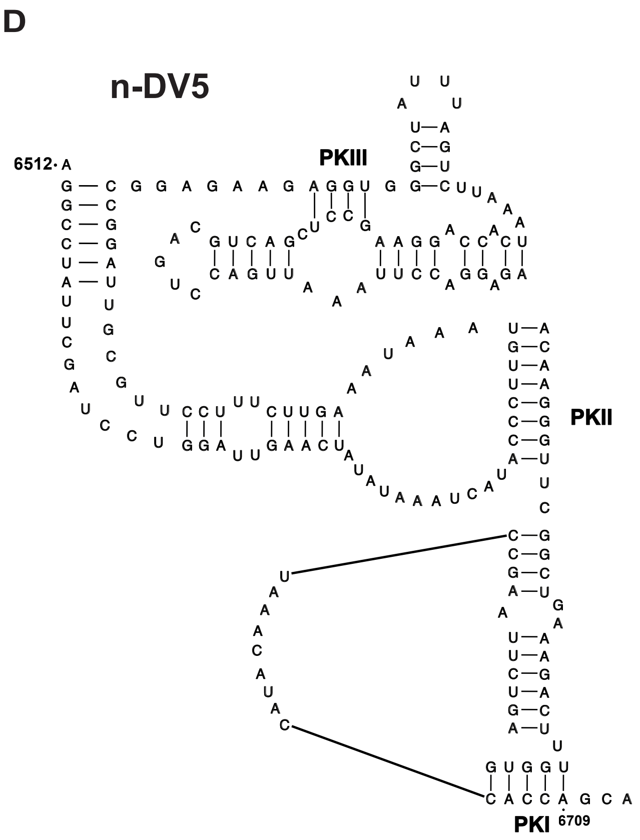
D
n-DV5
PKIII
PKII
PKI
6512
6709

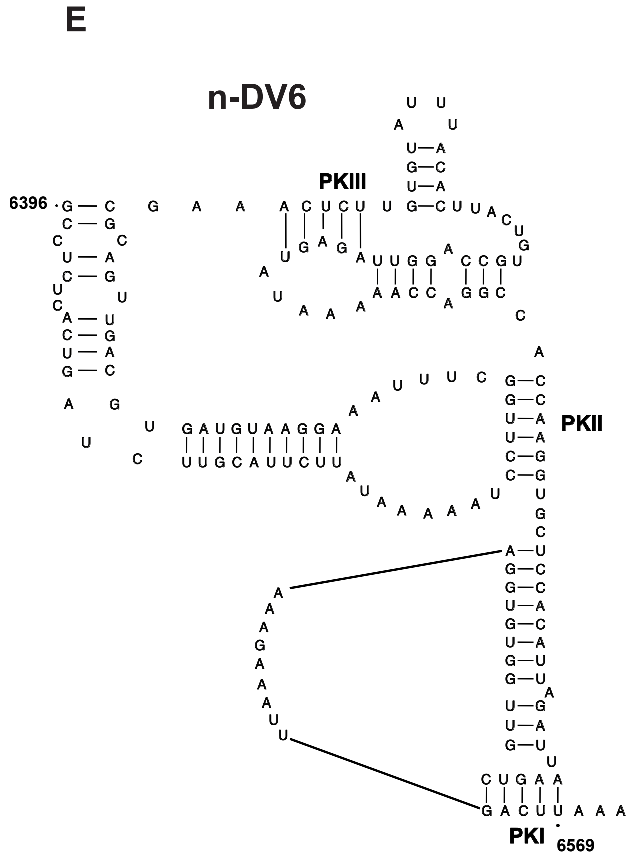
E
n-DV6
PKIII
PKII
PKI
6396
6569

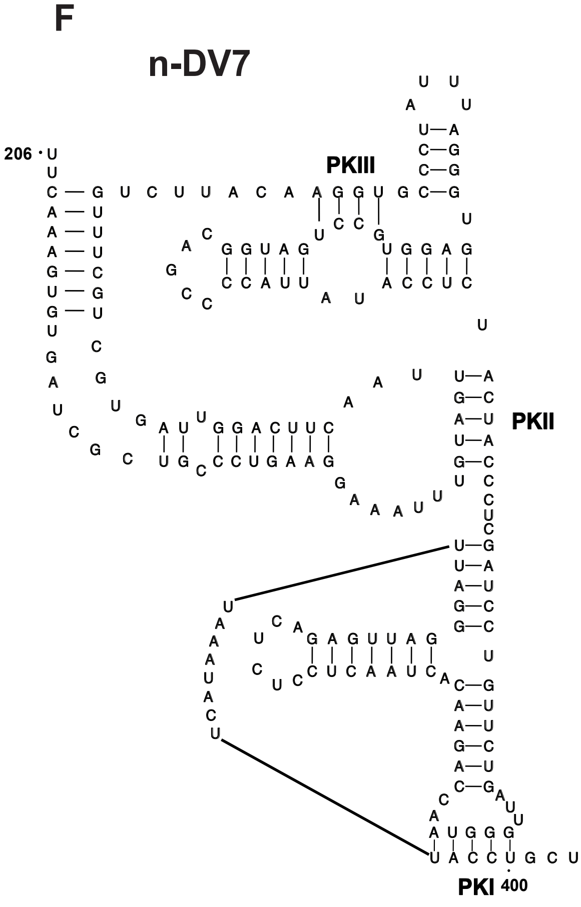
F
n-DV7
PKIII
PKII
PKI
206
400

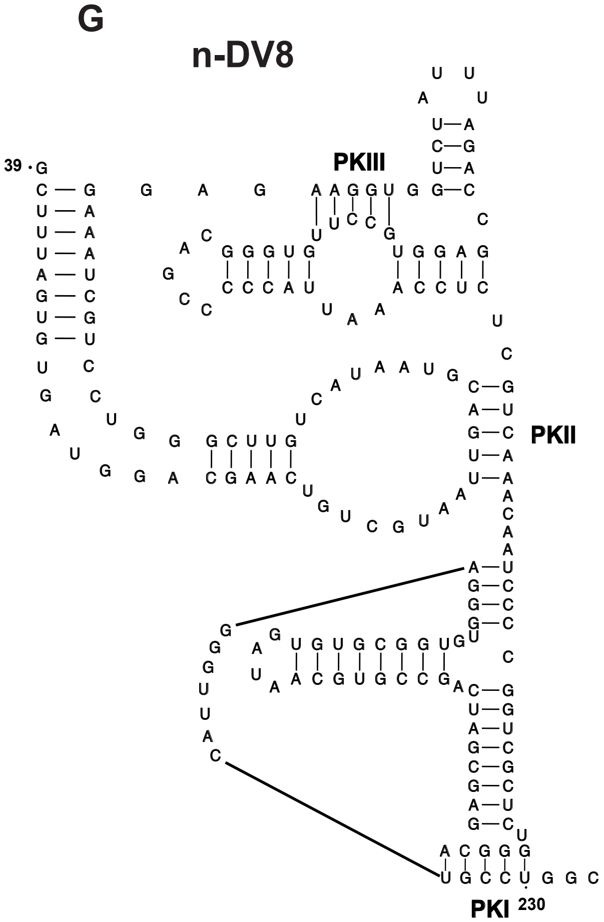
G
n-DV8
PKIII
PKII
PKI
39
230

A

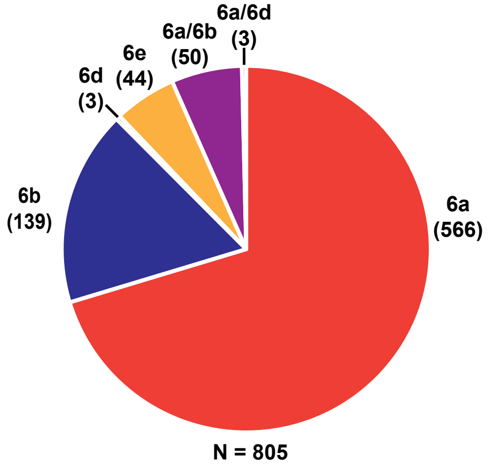

B

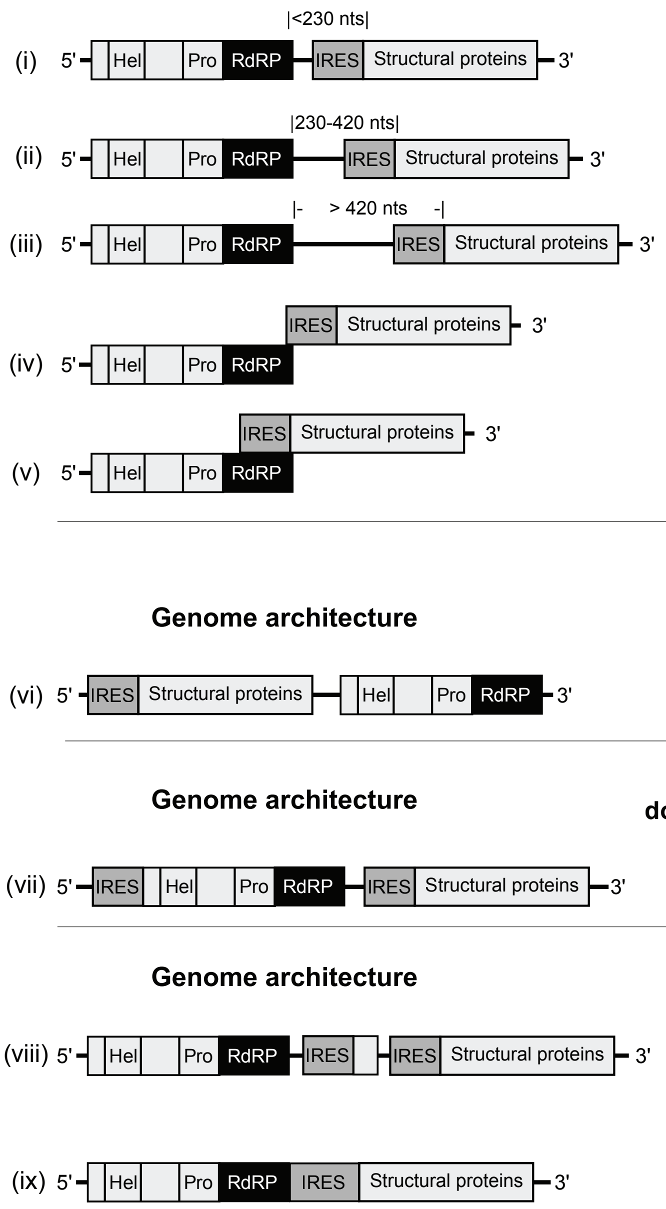

Type IRES

Genome architecture	6a	6b	6c	6d	6e	6f	6a/6b	6a/6e	6a/6d	Total
(i)	225	99	-	-	36	-	16	3	-	379
(ii)	80	9	-	-	-	-	12	1	1	103
(iii)	47	2	-	-	-	-	-	-	-	49
(iv)	21	6	-	-	3	-	11	-	-	41
(v)	1	-	-	-	-	-	-	-	-	1

Type IRES

Genome architecture	6a	6b	6c	6d	6e	6f	6a/6b	6a/6e	6a/6d	Total
(vi)	-	5	-	-	-	-	6	-	-	11

Genome architecture	upstream IRES: 6a<br>downstream IRES: 6a	6b<br>6a	6a<br>6a/6b	6a/6b<br>6a/6b	Total
(vii)	7	-	-	2	9

Genome architecture	6a 6a	6d 6d	6a 6d	6d 6a	6ab 6b	6ab 6ab	6b 6b	Total
(viii)	1	-	1	-	6	-	-	8

Genome architecture	6a	6b	6c	6d	6e	6f	6ab	6ae	6ad	Total
(ix)	10	-	-	-	-	-	-	-	-	10

Figure S3. A. Distribution of unique Type 6 IRESs in genomes >6Kb. **B**. Diversity of genome architecture arrangements and distribution of annotated Type 6 IRESs

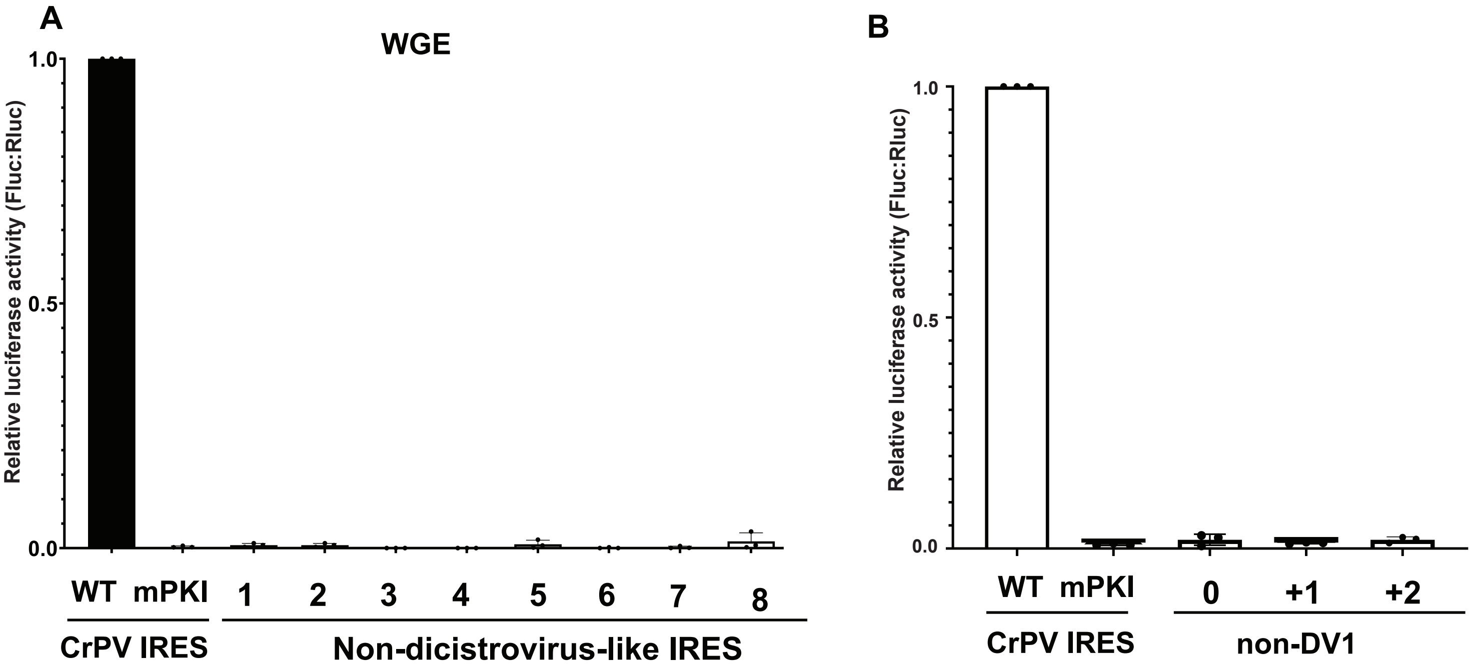

Figure S4. (**A**) Translational activity of Non-dicistrovirus IGR RNA candidates in wheat germ extracts (WGE) **(B)** Tranlsational activity of non-DV-1 IGR RNA in 0, +1 and +2 reading frames. Shown are averages ± s.d. from at least three independent experiments.

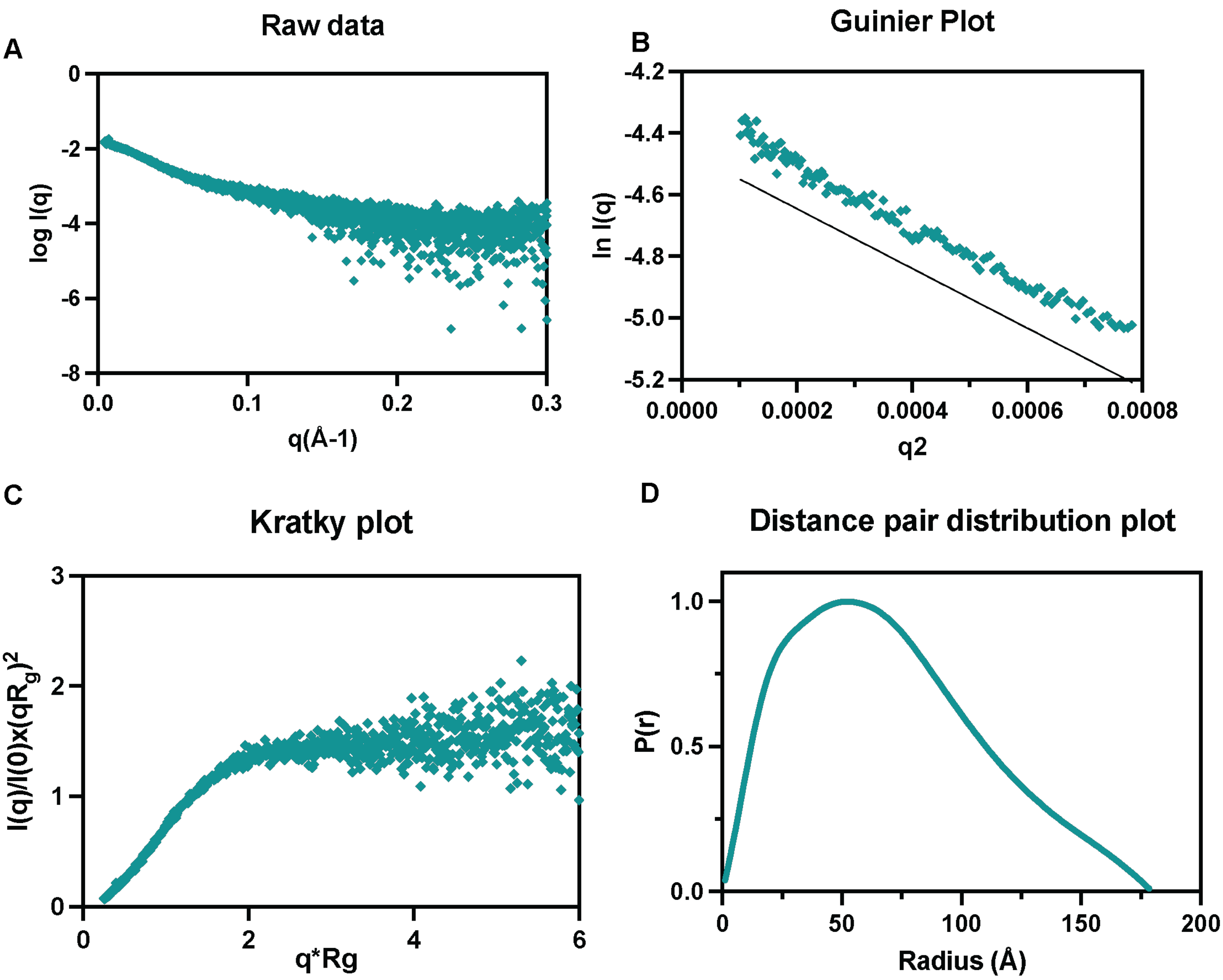

E

Models	NSD	Correlation coefficient
tombv1_cg-001605_AAr.pdb	1.078	0.7398
tombv1_cg-001707_AAr.pdb	1.063	0.7268
tombv1_cg-001713_AAr.pdb	1.039	0.7462
tombv1_cg-001716_AAr.pdb	1.049	0.7336
tombv1_cg-001868_AAr.pdb	1.070	0.7749
tombv1_cg-001904_AAr.pdb	1.084	0.7299

F

RNA	Guinier R_g (Å)	P(r) R_g (Å)	MW (KDa)	D_{max} (Å)	χ^2	NSD
TombV1	55.84 ± 0.06	56.07 ± 0.02	~ 108.3	179.6	~1.07	1.00 ± 0.03

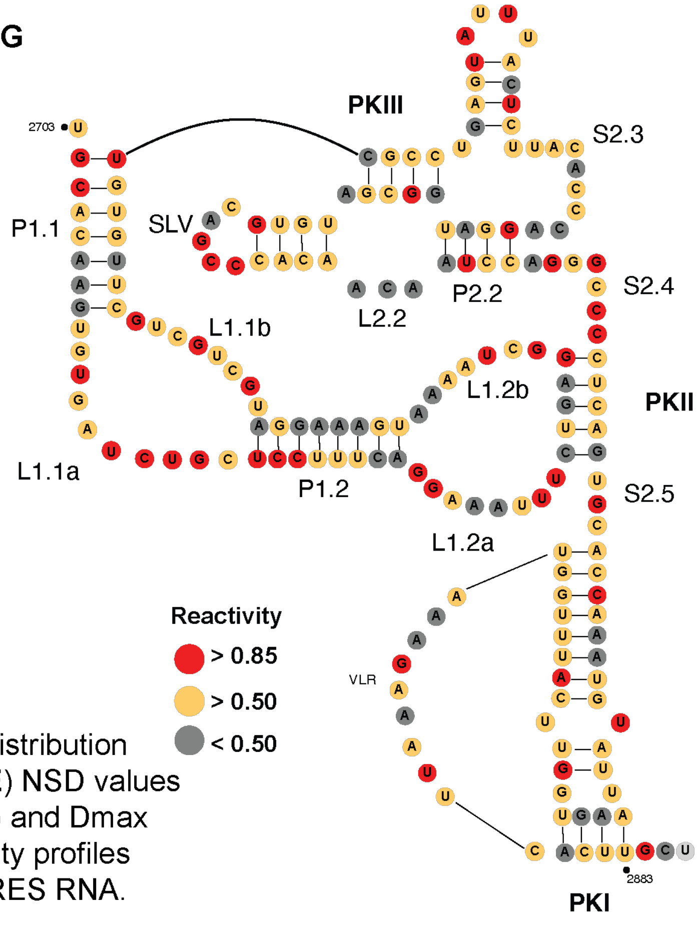

Figure S5. A-D) Raw data (Guinier, Kratky and pairwise-distribution plots) for non-DV1 RNA prodduced from SAXS analysis. **E**) NSD values and correlation coefficient for different models. **F**) Rg, P(r) and Dmax values for the analysed non-DV1 RNA. **G)** SHAPE-reactivity profiles of nucleotides mapped onto the predicted non-DV1 IGR IRES RNA.

Extended Data

SAXS analysis of non-DV-1 IRES

SAXS is a biophysical method that offers low-resolution structural information about the shape and conformation of RNA in solution [1,2]. The raw data for non-DV-1 RNA (Fig. S5A-S5D) produced Guinier, Kratky, and P(r) plots. The Guinier analysis was applied to evaluate the quality of the collected data and determine the radius of gyration (R_g) (Fig. S5D). The plots were subjected to linear regression fitting, and the observed linearity indicates that the samples are devoid of aggregation and are monodisperse (Fig. S5B), with an R_g of approximately 55.84 Å. We then used a dimensionless Kratky analysis to assess the compactness of each molecule. Upon reaching a near-maxima on the dimensionless Kratky distribution, the data set exhibits a plateau-like distribution (Fig. S5C), signifying an extended conformation for the RNAs. Finally, we constructed a paired electron distribution function to calculate reciprocal-space R_g and their maximum dimensions (D_{max}). The R_g calculated from the P(r) function (56.07 Å) agrees with Guinier R_g (55.84 Å). Also, the shape of the P(r) curves from symmetrical Gaussian distributions supports the Kratky analysis, indicating that an elongated molecule is under examination (Fig. S5D). Furthermore, it is evident from the P(r) that the non-DV-1 RNA has a D_{max} of approximately 175 Å. DAMMIN was employed to perform ab initio modelling and produce low-resolution envelope structures for non-DV-1 RNAs using the P(r) information.

References

1. D'Souza MH, Mrozowich T, Badmalia MD, Geeraert M, Frederickson A, Henrickson A, et al. Biophysical characterisation of human LincRNA-p21 sense and antisense Alu inverted repeats. Nucleic Acids Res. 2022;50: 5881–5898. doi:10.1093/nar/gkac414

2. Chen Y, Chapagain S, Chien J, Pereira HS, Patel TR, Inoue-Nagata AK, et al. Factor-Dependent Internal Ribosome Entry Site and -1 Programmed Frameshifting Signal in the Bemisia-Associated Dicistrovirus 2. Viruses. 20240428th ed. 2024;16. doi:10.3390/v16050695
